# Supplementary material for: Postoperative Electroacupuncture Boosts Cognitive Function Recovery after Laparotomy in Mice
Source: Biomolecules. 2024 Oct 10;14(10):1274. doi: 10.3390/biom14101274 (PMC11506768; doi:10.3390/biom14101274)
Supplement: Supplementary file 1 [file biomolecules-14-01274-s001.zip › biomolecules-3164713-supplementary.pdf]

Supplementary File

IL-1β	F: GATGAAGGGCTGCTTCCAAAC<br>R: TCCACAGCCACAATGAGTGA
IL-8	F: TGCCGTGACCTCAAGATGTGCC<br>R: CATCCACAAGCGTGCTGTAGGTG
IL-6	F: TTCACAAGTCCGGAGAGGAG<br>R: TCCACGATTTCCCAGAGAAC
IL-10	F: CCAAGCCTTATCGGAAATGA<br>R: TTCTCACCCAGGGAATTCAA
TNFα	F: CCCCAGTCTGTATCCTTCT<br>R: ACTGTCCCAGCATCTTGT
MCP-1	F: AGCTGTAGTTTTTGTCACCAAGC<br>R : GACCTTAGGGCAGATGCAGT
GAPDH	F: ATTCAACGGCACAGTCAA<br>R: CTCGCTCCTGGAAGATGG

Supplementary table S1. The primer sequences used in real-time PCR.

Target protein	Dilution	Resource	Catalog no.
AT180 (pThr^{231}/Ser^{235})	1:1000	Invitrogen, Thermo Fisher Scientific, Carlsbad, CA, USA	MN1040
AT8 (pSer^{202}/Thr^{205})	1:1000	Invitrogen, Thermo Fisher Scientific, Carlsbad, CA, USA	MN1020
pS404 (pSer^{404})	1:3000	Invitrogen, Thermo Fisher Scientific, Carlsbad, CA, USA	44-758G
pS404 (pSer^{396})	1:3000	Invitrogen, Thermo Fisher Scientific, Carlsbad, CA, USA	44-752G
Pan tau (polyclonal rabbit anti-human tau)	1:30,000	DAKO, Glostrup, Denmark	A0024
Synapsin-1	1:10,000	Cell signaling Technology, Danvers, MA, USA	5297
Synaptophysin	1:30,000	Thermo Fisher Scientific, Carlsbad, CA, USA	PA1-1043
NMDA Receptor 2B (GluN2B)	1:2000	Cell signaling Technology, Danvers, MA, USA	4207
Phospho-GSK-3β (Ser^{9})	1:1000	Cell signaling Technology, Danvers, MA, USA	9336S
Glycogen synthase kinase-3β (GSK-3β)	1:1000	Cell signaling Technology, Danvers, MA, USA	9315S
Phospho-SAPK-JNK (Thr^{183}/Tyr^{185})	1:1000	Cell signaling Technology, Danvers, MA, USA	9251S
Stress-activated protein kinases (SAPK)/c-Jun N-terminal kinase (JNK)	1:3000	Cell signaling Technology, Danvers, MA, USA	9258S
phospho-Jak2 (Tyr$^{1007/1008}$)	1:1000	Cell signaling Technology, Danvers, MA, USA	3771S
Jak2	1:1000	Cell signaling Technology, Danvers, MA, USA	3230S
Phospho-Stat3 (Tyr^{705})	1:1000	Cell signaling Technology, Danvers, MA, USA	9131S
Stat3	1:1000	Cell signaling Technology, Danvers, MA, USA	9139S
α-tubulin	1:40,000	Sigma-Aldrich, St. Louis, MO, USA	T9026
Glyceraldehye-3-phosphate dehydrogenase (GAPDH)	1:3000	Sigma-Aldrich, St. Louis, MO, USA	G8795
β-actin	1:30,000	Sigma-Aldrich, St. Louis, MO, USA	A5441

Supplementary Table S2. Primary antibodies used in Western blot analysis.

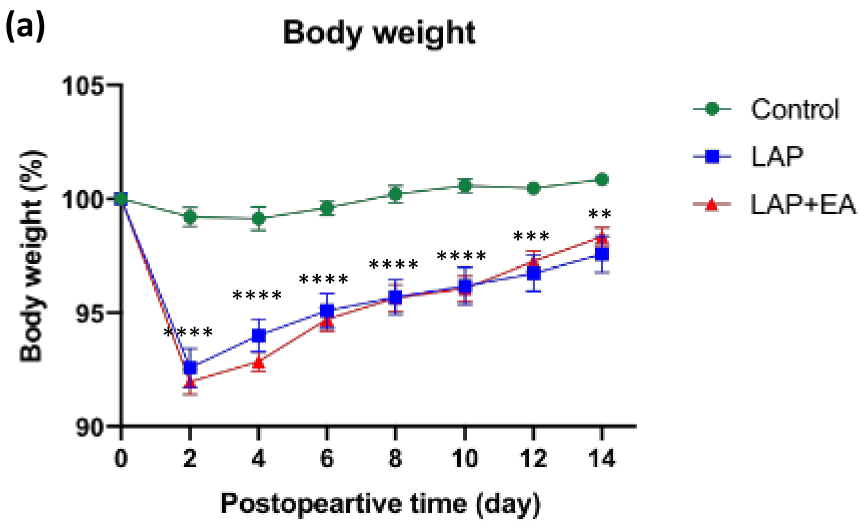

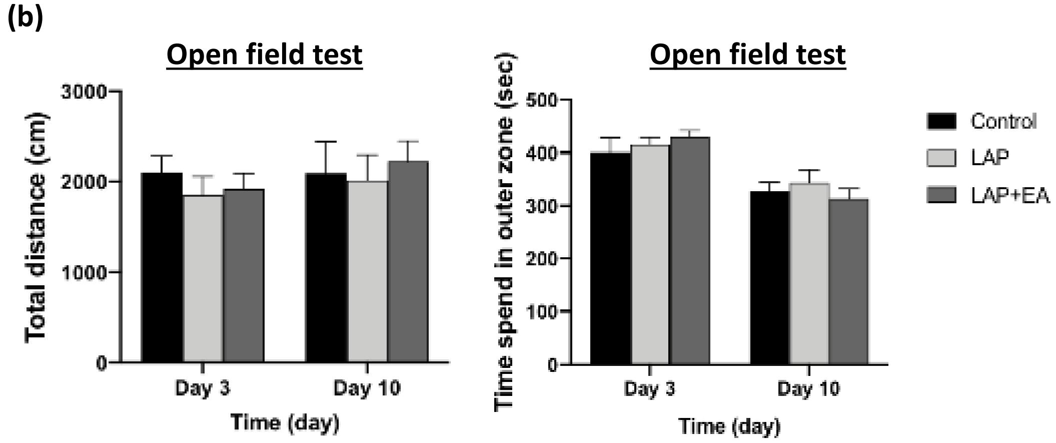

Supplementary figure S1. Body weight, general locomotor activity levels, and anxiety- or depression-like behaviors. (a) Body weight as a percentage of the baseline was analyzed using two-way ANOVA followed by Bonferroni's post-hoc test. From days 0 to 14, the body weights of mice in the LAP and LAP+EA groups were all significantly lower than those of the control group. $^{**}p < 0.01$, $^{***}p < 0.001$, $^{****}p < 0.0001$, compared to control. n = 8-10. (b) The open field test was conducted on days 3 and 10 to evaluate general locomotor function and anxiety-like behavior. The total distance travelled indicated general locomotor function, and the time spent in the outer zone indicated anxiety-like behavior. No significant differences were found between the three groups in these tests. Data were analyzed using a two-way ANOVA followed by Tuekey's post-hoc test. n = 8-10 for the open field test.

Body weight

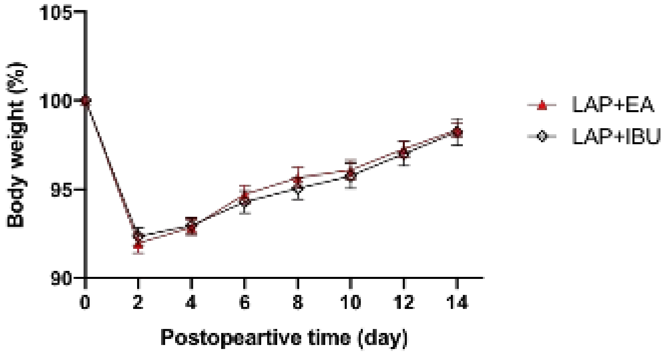

Supplementary figure S2. Body weight between the EA and ibuprofen group. Body weight as percentage of baseline were analyzed using two-way ANOVA followed by Bonferroni's post-hoc test. From Day 0 to 14, the body weight of mice in the EA group and ibuprofen group were similar. $n = 8$.

(a) Plasma-7 days **(b) Plasma-14 days**

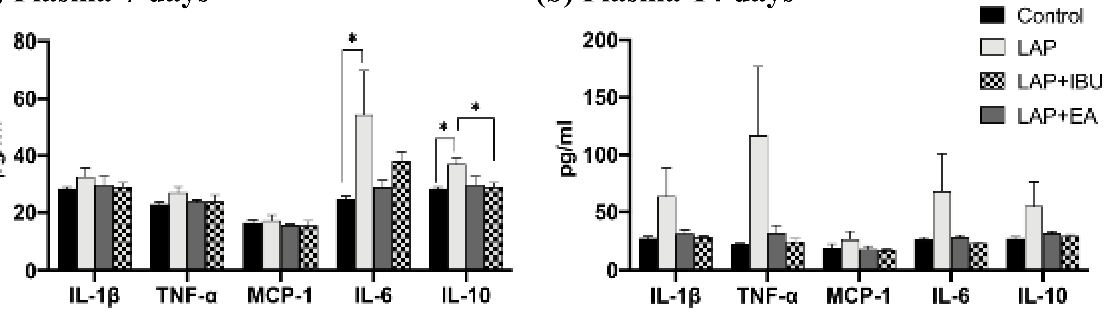

Supplementary figure S3. Changes in peripheral cytokine levels after laparotomy. Systemic inflammation was investigated by the detection of inflammatory cytokines in the plasma using the Milliplex assay (a) 7 days and (b) 14 days after laparotomy. Data were analysed using one-way ANOVA followed by Bonferroni's post-hoc test. n = 8–10; *$p < 0.05$.
